# Setting priorities for humanitarian water, sanitation and hygiene research: a meeting report

**DOI:** 10.1186/s13031-018-0159-8

**Published:** 2018-06-15

**Authors:** Lauren D’Mello-Guyett, Travis Yates, Andy Bastable, Maysoon Dahab, Claudio Deola, Caetano Dorea, Robert Dreibelbis, Timothy Grieve, Thomas Handzel, Anne Harmer, Daniele Lantagne, Peter Maes, Melissa Opryszko, Sarah Palmer-Felgate, Brian Reed, Rafael Van Den Bergh, Dominique Porteaud, Oliver Cumming

**Affiliations:** 1 Department for Disease Control, Faculty of Infectious and Tropical Diseases, London School of Hygiene and Tropical Medicine, London, UK; 2 Independent Consultant, Boston, MA USA; 3 Oxfam International, Oxford, UK; 4 Elrha, London, UK; 5 Save the Children UK, London, UK; 6 Department of Civil Engineering, University of Victoria, Victoria, British Columbia Canada; 7 UNICEF, New York, USA; 8 Emergency Response and Recovery Branch, Center for Global Health, Division of Global Health Protection, Centers for Disease Control and Prevention, Atlanta, GA USA; 9 Department of Civil and Environmental Engineering, Tufts University, Boston, MA USA; 10 Water, Hygiene and Sanitation Unit, Medical Department, Médecins Sans Frontières- Operational Center Brussels, Brussels, Belgium; 11 Office of U.S. Foreign Disaster Assistance, United States Agency for International Development, Washington D.C., USA; 12 Water, Engineering and Development Centre, Loughborough University, Loughboroughg, UK; 13 Operational Research Unit (LuxOR), Médecins Sans Frontières -Luxembourg, Luxembourg, Luxembourg; 14 Global WASH Cluster, UNICEF, Geneva, Switzerland; 15 Independent Consultant, London, UK

## Abstract

Recent systematic reviews have highlighted a paucity of rigorous evidence to guide water, sanitation and hygiene (WASH) interventions in humanitarian crises. In June 2017, the Research for Health in Humanitarian Crises (R2HC) programme of Elrha, convened a meeting of representatives from international response agencies, research institutions and donor organisations active in the field of humanitarian WASH to identify research priorities, discuss challenges conducting research and to establish next steps. Topics including cholera transmission, menstrual hygiene management, and acute undernutrition were identified as research priorities. Several international response agencies have existing research programmes; however, a more cohesive and coordinated effort in the WASH sector would likely advance this field of research. This report shares the conclusions of that meeting and proposes a research agenda with the aim of strengthening humanitarian WASH policy and practice.

## Introduction

Over the last decade, numerous scoping and systematic reviews have concluded that water, sanitation and hygiene (WASH) interventions in humanitarian crises can yield important health and social benefits for vulnerable affected populations [1–8]. However, all reviews found that: i) the current evidence base supporting humanitarian WASH interventions is limited; ii) policy and practice is often based on operational experience rather than independent evaluation; iii) that research to date has been dominated by studies of household or point-of-use (POU) water treatment with little research on the health or social impacts of community level water supply, sanitation, and hygiene interventions, or relative benefits of combined WASH interventions; and iv) evidence generation in humanitarian crises remains challenging and ad hoc.

This paucity of evidence is reflected in guideline recommendations for humanitarian WASH programmes, such as the SPHERE standards [9], which are often not supported by rigorous, published evidence [9–15]. Although many response agencies routinely conduct programme evaluations, these are often not rigorous nor published; thereby limiting the uptake of findings. While this issue is common across the humanitarian sector, a systematic review of all public health interventions in humanitarian crises published in 2017, found that WASH interventions were supported by far fewer studies (*n* = 6) than other areas of public health [5].

In November 2016, during the 7th Emergency Environmental Health Forum (EEHF) in Kathmandu, Nepal [16], a group of leading response agencies, research institutions and donor organisations raised the idea of conducting a research priority-setting exercise for humanitarian WASH. In response, Elrha’s Research for Health in Humanitarian Crises (R2HC) programme convened a two-day meeting (June 29-30th 2017) in Windsor, United Kingdom, with a group of international response agencies, research institutions, and donor organisations (Appendix A) [17].

The objective of the meeting was to identify key public health research priorities for humanitarian WASH research, informed by response agency, research institution and donor organisation perspectives, to strengthen policy and practice. Momentum for the meeting was supported by concurrent funding initiatives for health in humanitarian crises research such as the R2HC, the Humanitarian Innovation Fund (HIF) and the Humanitarian Evidence Programme (HEP). We have published this report of the meeting to expand the discussion beyond those who attended and to stimulate much-needed research interest and investment in the field of humanitarian WASH.

## Key conclusions

The main outcomes of the meeting are presented below under three sub-headings: Research Priorities for Humanitarian WASH; Challenges with Conducting Research in Humanitarian Crises and Moving the Humanitarian WASH Research Agenda Forward.

### Research priorities for humanitarian WASH

Ten key research priorities were identified by the attendees reflecting operational, research and donor perspectives:

1. **WASH for the prevention and control of cholera**
  To understand the relative importance of cholera transmission in the domestic and public domains and implications for effective humanitarian WASH responses
  To rigorously assess the effectiveness of conventional WASH interventions on cholera transmission in humanitarian crises
2. **Menstrual hygiene management (MHM)**
  To identify effective culturally appropriate MHM interventions for women and girls affected by humanitarian crises
  To identify social and cultural barriers to safe MHM interventions among women and girls affected by humanitarian crises
  To design and test strategies to integrate MHM interventions for different response phases (e.g. acute and post-acute phase)
3. **Coordination of Oral Cholera Vaccine (OCV) and WASH interventions**
  To evaluate the relative effectiveness of combined WASH and OCV versus OCV alone on cholera transmission in different settings and for different populations (e.g. in endemic versus epidemic settings)
  To design and test strategies for coordinating and targeting WASH and OCV interventions in different settings and for different populations (e.g. in endemic versus epidemic settings)
4. **Strengthening the design and targeting of hygiene kits in WASH programmes**
  To determine the most appropriate products to be included in hygiene kits in different responses phases (e.g. acute and post-acute phase)
  To understand the utilisation of hygiene kits and evaluate their effectiveness to control infectious disease outbreaks (e.g. cholera, Ebola, Hepatitis E)
5. **WASH in moderate/severe acute malnutrition programmes**
  To identify appropriate WASH interventions to include in programmes for management of child moderate acute malnutrition (MAM) and severe acute malnutrition (SAM) in humanitarian crises
  To evaluate the effect of combined WASH and MAM/SAM interventions on reduce relapse rates, duration of treatment and overall mortality among children in humanitarian crises
6. **WASH-related enteric disease and transmission**
  To ascertain aetiology of childhood diarrhoeal disease in humanitarian settings and other symptomatic and asymptomatic consequences of faecal oral diseases
  To assess dominant transmission routes for specific diarrhoeal diseases (e.g. cholera, *Shigella*, pathogenic *E. coli*) which present a high burden in given humanitarian crises
7. **WASH for reproductive, maternal and neonatal health**
  To design humanitarian WASH interventions that support safe delivery practices at home and in facilities
  To evaluate whether humanitarian WASH interventions at home and in facilities can improve maternal and neonatal health outcomes
8. **Strengthening the hygiene component of humanitarian WASH programmes**
  To understand the motivational drivers of sustained handwashing practices and maintenance of facilities in humanitarian crises
  To design and evaluate behaviour change interventions to increase handwashing with soap at key times in humanitarian crises
  To design and evaluate behaviour change interventions that improve the sustainability of other hygiene behaviours (e.g. personal and domestic hygiene) in populations affected by humanitarian crises
9. **Strengthening the sanitation component of humanitarian WASH programmes**
  To design and evaluate behaviour change interventions to increase sanitation uptake in humanitarian crises
  To design and evaluate novel sanitation technologies for use in the acute phase of humanitarian crises
  To design and evaluate novel technologies and approaches to improve sanitation uptake among vulnerable populations, including people with disabilities and young children, in humanitarian crises
10. **WASH as part of the effective transition between emergency and development**
  To evaluate the impact of strategies for improved coordination amongst WASH response agencies and between WASH and health sectors on performance indicators
  To describe and evaluate current coordination mechanisms for transitionary handover of WASH services from response agencies to national government and/or other development actors

### Challenges with conducting research in humanitarian crises

Participants shared their current research programmes and related how this work had been initiated and developed. A wide range of research activities were shared but these efforts were largely independent and uncoordinated, with topics reflecting individual organisation’s mandates rather than broader strategic or sectoral areas of interest or concern. Participants remarked that it might not be feasible or even desirable for all agencies to adhere to a common research agenda due to organisational priorities, although a common agenda might help to guide future research activities, donor investments and research collaborations.

Humanitarian crises, by definition, provide a challenging context for undertaking research. Most often in crises, affected populations have acute needs, response agencies operate under severe logistical constraints, and the socio-political environment is often unstable. Resources and opportunities for research in these settings are limited, particularly more intensive research involving differing standards of care and experimental interventions. Organisations undertaking research need to plan in advance, often significantly ahead of an actual emergency yet remain pragmatic and adaptive when research is executed.

#### Participants identified a set of key barriers to conducting research

- Difficult to secure ethical approval rapidly
- Limited investment in research and resources not readily available
- Challenges in reconciling interests and needs of response agencies and research institutions with regard to what constitutes “sufficient rigour”
- Conflicting timelines of response efforts and research protocols
- Lack of staff trained in research methods within humanitarian organisations
- Weak connections between research, and policy and practice functions within agencies
- Limited public data sharing, and publication of internal evaluations

### Moving the humanitarian WASH research agenda forward

There was consensus on the importance of research and the need to strengthen the evidence base for these interventions but also a recognition that dedicated efforts are needed to support uptake of findings within and across sector agencies.

Many of the response agencies present had specific programmes to train staff and increase research outputs. Examples included the Centers for Disease Control and Prevention (CDC) “Epidemiology for WASH” training programme, the “Using evidence for WASH policy and practice” course run between UNICEF and the London School of Hygiene and Tropical Medicine (LSHTM) and other WASH-specific training programmes run internationally [18], the Médecins Sans Frontières (MSF) operational research trainings “SORT-IT” [19] and recent “WASH-IT” spin off, and dedicated operational research units such as those within Action Contre La Faim (ACF), MSF and among other agencies. There were also examples of academic-humanitarian partnerships with universities in the U.S., UK, Europe, Africa and Asia conducting collaborative research with response agencies; often involving both Masters (MSc) and Doctoral (PhD) students.

A notable challenge remains in establishing and maintaining connections from research findings and teams operating in-country. The group recognised that research uptake needs careful consideration in dedicated training programmes in order to build local capacity for research and evaluation and as an advocacy tool across the humanitarian WASH, health, nutrition and shelter sectors. Revisions to guidelines and manuals needs to be evidence-based with Specific, Measurable, Achievable, Relevant and Time-Related (SMART) indicators to aid monitoring and evaluation. Participants noted that a cultural shift is required within most organisations to carry out and incorporate research within their programmes or to build linkages with agencies who have that technical expertise.

Engagement of all members of the Global WASH Cluster was flagged as important to establish a representative view and ensure the involvement of the broader group of those involved in the humanitarian WASH response. This meeting was intended to gauge interest and undertake initial discussions on the direction for future WASH in humanitarian crises research. The group made two commitments towards sustaining momentum on this agenda:

1. **Engage with the wider humanitarian WASH community:** A survey will be undertaken amongst response agencies with active WASH programmes to map the current and future research activities and to identify research priorities. Full engagement with field staff as well as international managers is necessary to foster buy-in and identify relevant research objectives. Immediate opportunities to broaden the dialogue on this agenda are the 2018, and ongoing editions of, the Emergency Environmental Health Forum (EEHF), the Water Engineering Development Centre (WEDC), and the University of North Carolina (UNC) Water and Health conferences.
2. **Knowledge Sharing:** Greater efforts will be made to disseminate publications, advertise training opportunities, publicise relevant calls for proposals and share information on ongoing research activities. Identified opportunities for this include the nascent Technical Working Group (TWiG) within the Global WASH Cluster and the use of existing repositories for published and non-published research outputs including those within the Global WASH Cluster platform [20] and others [21, 22].

## Conclusion

The scale, duration and complexity of humanitarian crises is increasing [23], with a growing population of highly vulnerable people. Responding to these crises requires strong evidence on what works to guide more effective and efficient investment and to achieve better health and social outcomes. Addressing the humanitarian WASH evidence gap is a joint concern of the response agencies, research institutions and donor organisations who attended this meeting. A greater commitment within the humanitarian WASH sector is needed to generate this evidence and an increased investment of resources to achieve this. Our meeting provided a platform for the leading international response agencies, research institutions and donor organisations active in the humanitarian WASH sector to undertake a participatory research priority setting exercise. We hope that sharing the conclusions from this meeting will support a broader and ongoing discussion in the humanitarian WASH sector as to how we can strengthen the evidence base to support the delivery of better WASH interventions for vulnerable crisis-affected populations.

## Appendix A

### Organisations represented

- Action Contre la Faim (ACF)
- Centers for Disease Control and Prevention (CDC)
- Elrha
- Global WASH Cluster (GWC)
- London School of Hygiene and Tropical Medicine (LSHTM)
- Médecins Sans Frontières (MSF)
- Office of U.S Foreign Disaster Assistance (OFDA), United States Agency for International Development (USAID)
- Oxfam
- Save the Children
- Tufts University
- UNICEF
- University of Victoria
- Water, Engineering and Development Centre (WEDC), Loughborough University

## Abbreviations

ACF: Action Contre la Faim
CDC: Centers for Disease Control and Prevention
DFID: Department for International Development
EEHF: Emergency Environmental Health Forum
HEP: Humanitarian Evidence Programme
HIF: Humanitarian Innovation Fund
LSHTM: London School of Hygiene and Tropical Medicine
MAM: Moderate Acute Malnutrition
MHM: Menstrual Hygiene Management
MSF: Médecins Sans Frontières
OCV: Oral Cholera Vaccine
POU: Point-Of-Use water treatment
R2HC: Research for Health in Humanitarian Crises
SAM: Severe Acute Malnutrition
SMART: Specific, Measurable, Achievable, Relevant and Time-Related
TWiG: Technical Working Group
UNC: University of North Carolina
WASH: Water, Sanitation and Hygiene
WEDC: Water, Development and Engineering Centre
